# Understanding physical activity from a cultural-contextual lens

**DOI:** 10.3389/fpubh.2023.1223919

**Published:** 2023-08-04

**Authors:** Carielle Joy Rio, Leorey N. Saligan

**Affiliations:** Division of Intramural Research, National Institute of Nursing Research, National Institutes of Health, Bethesda, MD, United States

**Keywords:** physical activity, healthy lifestyle, culture, health promotion, exercise

## Abstract

This paper aims to emphasize the need to acknowledge unique cultural and contextual meanings of physical activity to improve health outcomes in different communities. Leininger’s Sunrise Model was used as the theoretical base to understand the complex cultural and contextual factors that influence physical activity. Beliefs and practices surrounding physical activity are influenced by a variety of cultural and contextual factors. Providing culturally relevant contexts to the meaning of physical activity allows opportunities for improving policies or programs that would engage individuals and communities in physical activity in culturally meaningful ways. Incorporating cultural and contextual factors is critical to promote physical activity, especially in minority and vulnerable communities.

## 1. Factors that influence physical activity

Physical activity has been found to be effective in preventing chronic diseases, including cardiovascular, respiratory, neurological, metabolic, psychiatric, and musculoskeletal diseases and certain types of cancer (1). The weekly recommended physical activity for adults is 150 minutes of moderate intensity activity or 75 minutes of vigorous intensity activities (2). Current recommendations of specific types of activities promoted in publicly-available sites such as the Presidential Active Lifestyle Award (3), the Physical Activity Guideline for Americans 2nd edition (4), and the physical activity health education materials from the National Institute of Aging website (5) do not include unique culturally-embraced notions of physical activity nor do they include social contexts to promote physical activity engagement, such as the presence of social and built-environmental factors in vulnerable communities.

This paper addresses the lack of culturally tailored physical activity guidelines by describing exemplars of unique cultural and contextual factors influencing physical activities. To undergird the discussion on a theory-based foundation, these exemplars are presented based on the social structures and cultural factors identified by the Leininger’s Sunrise Model (6). To our knowledge, this model has not been used to study physical activity in a specific population. The goal of this paper is to emphasize the need to acknowledge unique cultural and contextual meanings of health behaviors, such as physical activity, to improve health outcomes in different communities.

The Leininger’s Sunrise Model provided a theoretical base to comprehensively examine the social structures and cultural factors that drive a fuller understanding of the definition of physical activity across various settings. The Sunrise Model posits that there are seven factors such as (1) technology (2), religion and philosophy (3), kinship and social structures (4), cultural beliefs and practices (5), politics (6), economics and (7) education that influence people’s care meanings, health practices, and patterns.

### 1.1. Technological factors

There is strong evidence supporting the benefits of technology in promoting physical activity. Wearable technologies provide objective and accurate assessment of physical activities, allowing individuals to monitor their physical activities, and even socially engage with their own communities. Recent advances in these technologies address commonly identified barriers to physical activity including lack of motivation, knowledge, and social support (7). For example, positive reinforcement features enhance motivation for individuals to engage in physical activities (7). A cloud-based eHealth intervention was found to increase walking activities by an additional 39% in terms of daily steps among African American women (8). However, there are also studies which reported that technological advancement further widens the gaps in health disparities experienced by racially, ethnically, and/or economically disadvantaged groups (9). If social determinants are not addressed, the persistence of the digital divide will continue to prevent disadvantaged populations from experiencing the benefits of technology (9). Unfortunately, even in countries where technologies such as wearable devices are readily available, the acceptance and proper use of wearable devices remain a challenge (10). There is a need to create technologies that are easy-to-use to address some of the limitations caused by active aging such as the decline in visual or auditory functions (10, 11).

### 1.2. Religious and philosophical factors

There are contrasting findings on the role or influence of religion in physical activity. Some studies suggested that religion or religious activities influence physical activity. For example, a study that evaluated a dance program among Samoans found that the inclusion of prayer influenced participants’ commitment to the dance program (12). In a study that involved Muslim university students, several participants cited the teaching of the Prophet Muhammad as a significant factor that encouraged them to engage in physical activities. These activities are often cited as ordered by the Prophet Muhammad to include archery, swimming, and horse riding (13). A state-level partnership with faith communities in North Carolina, USA utilized faith communities as safe spaces where people can engage in physical activities by making faith community spaces accessible for physical activities (14). To encourage physical activity, faith communities have intentionally included breaks in between meetings to allow members to be physically active and have repaved parking lots to make them more suitable for walking. This partnership between the state of North Carolina and faith communities within the state resulted to an improvement in physical activity in 49% of the members of the participating faith communities (14).

However, there are also studies that found no correlation between religion and physical activity. A study that explored religiosity and physical activity among African Americans and Hispanic women using a 6-month Health is Power (HIP) intervention noted that religiosity is not a significant predictor of physical activity change (15). Another study that included adults from various religions (Islam, Christianity, Buddhism, and Hinduism) concluded that religion does not exert mediating effect on motivation to and actual participation in physical activities (16).

### 1.3. Family and kinship

Physical activity is influenced by interpersonal relationships at varying levels - family, organization, and community (17). Social norms regarding the acceptability of specific physical activities shape people’s attitudes and behavior. Social control and social support were identified as factors that influenced personal choices related to physical activity (18, 19). In a study conducted on a population from China, it was found that parents, especially fathers, are the main motivators of physical activities in children (20). However, families emphasized the importance of academic activities over sports or other forms of physical activity (21). The prevailing expectation on the role of women in the family, such as taking more active roles in childcare decreases the women’s sense of having the choice to control and regulate their physical activities (22).

Aside from the family, the wider cultural community also affects people’s engagement in physical activities. The opportunity to network with people from the same cultural background was also identified as a reason why people participated in physical activities (23). A study focusing on immigrants and refugees in Minnesota, USA noted that people were more motivated to be physically active when they knew someone in their community who had been successful in managing their personal obligations while remaining physically active (24). In contrast, solitary physical activities were viewed as an opportunity to distance oneself from social issues (17). Physical activity, whether done with others or in solitude, is affected by how people relate with others.

### 1.4. Cultural values and lifeways

Culturally constructed notions on gender appropriateness are the most cited cultural challenge surrounding physical activity (25). In a study that compared the level of physical inactivity in Arab countries, it was noted that women were generally less engaged in physical activities compared to men (26). In Muslim communities, females are often not permitted to exercise in public areas such as a gym or park where there is lack of gender segregation. In some instances, women are to be chaperoned when exercising in public facilities (13, 26).

Gender roles and gender expectations are significant aspects of cultural values and beliefs that shape people’s engagement in physical activity. For example, female Hispanic college students cited that cultural norms regarding appropriate activities for women were factors they considered when engaging in physical activities. Vigorous activities were generally considered to be unfeminine (27). Parents also tended to be more concerned about safety issues when their female children engaged in physical activities outside of their homes as compared to male children (28). Other aspects of gender norms, such as the dress codes for females that are observed by some cultures may not be suitable for certain physical activities (26).

### 1.5. Political and legal factors

There are limited studies on the role of politics and legal factors on physical activity. The role of policies and legislation in various aspects of health promotion such as regulation of tobacco use and alcohol consumption have been well established (29, 30). WHO recognizes that there is no “one size fits all” rule in designing policies to improve physical activity. The challenge is how interconnected factors can be aligned to ensure progress in the agenda of getting the population to be physically active (1). Despite the limited literature on the direct role of politics and legislation in physical activity engagement of a population, there are a growing number of studies that emphasize on the role of the built environment in promoting physical activity (13, 31, 32). It is noteworthy that the important aspects of designing built environments such as urban green space planning, land use, connectivity and pollution control require legislative action and creation of public policies (31).

### 1.6. Economic factors

A study involving 22 countries in Africa, most of which are classified as low-income countries, found that 79% of the population met the physical activity recommendation of the World Health Organization (WHO) (33). It was noted that although leisure time physical activity was minimal (5.3%), occupation-related physical activity accounted for almost half (48.6%) of the study population’s total physical activity (33). Focusing on leisure time physical activity when exploring the relationship between physical activity and economic conditions may lead to vague conclusions when other domains of physical activity are not accounted for (34). Also, understanding the role of economics in physical activity should be viewed not only in terms of wealth but also in terms of wealth gaps. Studies suggest that income disparity is a more significant predictor of physical activity engagement. A higher percentage of physically inactive individuals was noted in countries with high income disparity, regardless of the country’s wealth (35).

### 1.7. Educational factors

Education is an important social determinant of many aspects of health including life expectancy and health behaviors (36, 37). A study involving adult Finns noted that higher self-reported and device-measured physical activity were linked with higher education level (38). Similarly, a study that explored self-reported physical activity among Koreans observed that higher education was associated with adequate physical activity. A high average number of days engaging in specific physical activities including walking, flexibility exercises, and vigorous-intensity physical activities were noted in those with at least a post-graduate level of education. Those with middle-school level education were noted to be more engaged in moderate-intensity physical activities (39). Earlier studies concluded that highly educated individuals tended to be more knowledgeable regarding health and were more likely to practice health-promoting behaviors (40, 41). However, in the United States, a study reported that only 22% of the participants from the general population were aware that there are physical activity guidelines for Americans. When divided according to educational level, only 28% of the participants with at least 4 years of college education reported knowing about the physical activity guidelines (42). In another study involving Australian adults, almost all the participants (99.6%) acknowledged that physical activity is necessary to maintain health, but majority were not able to identify the recommended amount of physical activity (43). A study of women living in rural communities in Minnesota, USA also found that engagement remained low despite their high level of knowledge regarding physical activity (44). Thus, it is important to explore not only people’s knowledge of physical activity, but also their actual engagement in physical activity.

### 1.8. Environmental context

The availability of public spaces for exercise, such as sidewalks and parks, encourages people to be more active (13). In addition, people also value accessibility to areas where they can engage in recreational physical activities (18). Neighbourhood features including the presence of parks and streets that connect residential areas to shops also facilitate physical activities, particularly walking (13). In addition to physical infrastructures, people’s perspective of the environment, such as aesthetics and safety, are other important aspects to consider. For example, a systematic review showed that African American women may continue to be physically inactive despite the availability of public spaces due to fear of violence, crime, drugs, or unleashed dogs in the neighbourhood (45). The presence of crime or the fear of crime in the neighbourhood may affect people’s likelihood to engage in physical activities (19). Some reasons related to the environment that deter people from engaging in physical activities include drug activities in parks and wildlife on walking trails (18). Improvement in public utilities such as transportation was also found to be linked with physical activity. A study found that cities with good public transportation and a higher number of transport stops tended to improve physical activity among its residents (32).

### 1.9. Language

Evidence regarding the role of language in physical activity remains scarce. However, some available literature suggests that a language barrier is likely to have a negative impact on people’s physical activity. A study of refugees in Denmark noted language as one of the external demands identified as a barrier to being physically active (46). Studies that compared groups in terms of language spoken support the hypothesis that language proficiency or a language barrier was an influencer of physical activity. For example, a study on Asian Americans found that the language spoken at home is linked with physical activity. A higher prevalence of Asian Americans who met the WHO physical activity guidelines spoke at least some English at home (47). In another study that compared Spanish-speaking and English-speaking Hispanics, the authors found that the prevalence of insufficient physical activity was higher among Spanish-speaking Hispanics (48).

Language barrier contributes to physical inactivity in immigrant and minority populations. However, this factor should be understood in terms of the link between language barrier and socioeconomic conditions (46, 48). In the study that compared Spanish-speaking and English-speaking Hispanics, a majority of Spanish-speaking Hispanics had less than a high school education and in general had lower total household income than their English-speaking Hispanic counterparts (40, 41). Thus, it may be challenging to establish the direct relationship between language and physical activity.

### 1.10. Ethnohistory

Very little is known about how ethnohistory affects physical activity. Literature discussing ethnohistory in relation to physical activity is scarce and most studies on this topic examined physical activity from anthropological and archaeological perspectives. However, almost all the literature on ethnohistory presents the interrelatedness of socioeconomic factors affecting physical activity, as discussed in the previous sections. For example, a study suggested that the indigenous people of the Lambayeque region of Northern Peru were engaged in strenuous physical activities that led to a high prevalence of degenerative joint diseases during the colonial era (49). Archaeologic analysis showed that repetitive bending of joints and the use of the upper body were the common movements. These types of movements can be linked to cotton cultivation which dominated the colonial economy of that era (49). From this example, it can be gleaned that political and economic conditions, as well as social structures (e.g., the roles of colonizers and colonial subjects) influence physical activity.

In summary, providing culturally relevant contexts to the meaning of physical activity allows opportunities for improving policies or programs that would engage individuals and communities in physical activity in culturally meaningful ways. It is not only about the length of time or the intensity of physical activity, but also about how physical activity makes people feel and how the larger context affects their overall health. This is especially beneficial for populations who require culture and context-specific physical activity interventions such as religious minorities, gender diverse individuals, as well as racial, ethnic, and linguistically diverse communities.

## 2. Strength, limitations, and future perspectives

Leininger’s Sunrise Model is a useful tool in promoting a more comprehensive understanding of physical activity to include cultural and contextual factors that shape people’s beliefs and behavior towards physical activity engagement (see Table 1). There is a need to re-examine and possibly modify success indicators used to measure the effectiveness of physical activity interventions. To date, most of the studies on physical activity based their data on physiologic parameters gathered using wearable devices (such as Fitbits), accelerometers, and other similar devices. Also, there are very limited tools that capture and process data on the contextual and social drivers of physical activity. Consensus in the terminologies and definitions used in research, policies, and practice guidelines related to physical activity is also important. This paper is based on a targeted narrative review that was conducted to provide an overview of available literature on physical activity that link to any of the factors in Leininger’s Sunrise Model. Further reviews using more comprehensive and robust methods, such as a systematic review, is highly recommended. Studies that review the utility of the model in understanding physical activity in specific populations would also provide significant contributions in developing models of care that incorporate culturally appropriate physical activity interventions designed to meet the needs of vulnerable populations.

**Table 1:** Summary of salient points under the different factors that may influence physical activity indicated in Leigninger’s Sunrise Model.

Factors	Salient points
Technological	Technology improves monitoring physical activities and can be used to enhance motivation to be active. However, the digital divide prevents disadvantaged groups from experiencing the benefits of technology in improving physical activity.
Religious and Philosophical	There were contrasting findings as to the association between religion and physical activities.
Family and Kinship	Interpersonal relationships play a role in physical activity. The influence of family values on physical activity intersects with cultural values and perception of priorities (e.g., academic activities are more important)
Cultural Values, Beliefs, and Ways of Living	Perceived gender appropriateness of physical activities, safety of public spaces and dress codes are important cultural considerations in planning physical activity interventions.
Political and Legal	Improving the built environment to encourage physical activity is closely link with legislations and public policies. However, there are limited studies on the direct role of political and legal factors on physical activities.
Economic	Studies on economic conditions and physical activity ought to consider both leisure and occupational physical activities.
Educational	Understanding the role of education in physical activity should not only consider the level of formal education, but also the knowledge of individuals regarding physical activity.
Environmental context	Available public spaces, good transportation and high perceived safety of a neighborhood increases physical activity.
Language	Language barrier is linked with physical inactivity in immigrant and minority populations.
Ethnohistory	Archeological evidence suggests that even in acient societies, economic conditions and social structures influence physical activity.

## Author contributions

CR: drafted the concept, searched literature, summarized findings, and prepared manuscript. LS: drafted the concept, summarized findings, and prepared manuscript. All authors contributed to the article and approved the submitted version.

## Conflict of interest

The authors declare that the research was conducted in the absence of any commercial or financial relationships that could be construed as a potential conflict of interest.

## Publisher’s note

All claims expressed in this article are solely those of the authors and do not necessarily represent those of their affiliated organizations, or those of the publisher, the editors and the reviewers. Any product that may be evaluated in this article, or claim that may be made by its manufacturer, is not guaranteed or endorsed by the publisher.

## Author disclaimer

The content is solely the responsibility of the authors and does not necessarily represent the official views of the National Institutes of Health and/or the National Institute of Nursing Research.
